# Carbon nanotubes: properties, synthesis, purification, and medical applications

**DOI:** 10.1186/1556-276X-9-393

**Published:** 2014-08-13

**Authors:** Ali Eatemadi, Hadis Daraee, Hamzeh Karimkhanloo, Mohammad Kouhi, Nosratollah Zarghami, Abolfazl Akbarzadeh, Mozhgan Abasi, Younes Hanifehpour, Sang Woo Joo

**Affiliations:** 1 Department of Medical Biotechnology, Faculty of Advanced Medical Sciences, Tabriz University of Medical Sciences, Tabriz 5154853431, Iran; 2 Department of Medical Nanotechnology, Faculty of Advanced Medical Sciences, Tabriz University of Medical Sciences, Tabriz 5154853431, Iran; 3 School of Mechanical Engineering, Yeungnam University, Gyeongsan 712-749, South Korea; 4 Department of Physics, College of Science, Tabriz Branch, Islamic Azad University, Tabriz, Iran; 5 Drug Applied Research Center, Tabriz University of Medical Sciences, Tabriz, Iran

**Keywords:** Carbon nanostructures, Flexibility, Toxicity, Drug delivery, Nanotubes

## Abstract

Current discoveries of different forms of carbon nanostructures have motivated research on their applications in various fields. They hold promise for applications in medicine, gene, and drug delivery areas. Many different production methods for carbon nanotubes (CNTs) have been introduced; functionalization, filling, doping, and chemical modification have been achieved, and characterization, separation, and manipulation of individual CNTs are now possible. Parameters such as structure, surface area, surface charge, size distribution, surface chemistry, and agglomeration state as well as purity of the samples have considerable impact on the reactivity of carbon nanotubes. Otherwise, the strength and flexibility of carbon nanotubes make them of potential use in controlling other nanoscale structures, which suggests they will have a significant role in nanotechnology engineering.

## Review

### Introduction

**Figure 1:**
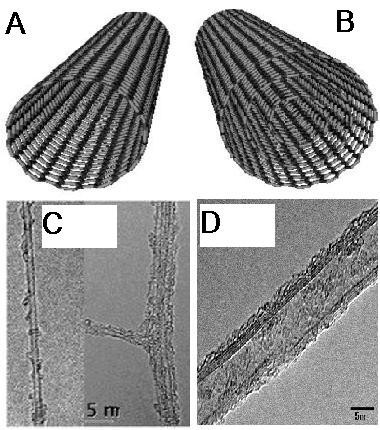
**Schematic structure and TEM images of SWCNT and MWCNT. (A)** Schematic structure of SWCNT and **(B)** MWCNT. The transmission electron microscope (TEM) images of a **(C)** SWCNT and **(D)** MWCNT [6-8].

Carbon is the chemical element with atomic number 6 and has six electrons which occupy 1 s^2^, 2 s^2^, and 2p^2^ atomic orbital. It can hybridize in sp, sp^2^, or sp^3^ forms. Discoveries of very constant nanometer size sp^2^ carbon bonded materials such as graphene [1], fullerenes [2], and carbon nanotubes [3] have encouraged to make inquiries in this field. Most of the physical properties of carbon nanotubes derive from graphene. In graphene, carbon atoms are densely organized in a regular sp^2^-bonded atomic-scale honeycomb (hexagonal) pattern, and this pattern is a basic structure for other sp^2^ carbon bonded materials (allotropes) such as fullerenes and carbon nanotubes. Carbon nanotube is theoretically distinct as a cylinder fabricated of rolled up grapheme sheet. It can divide into a single well or multiple wells. Nanotubes with single well are described as single-wall carbon nanotubes (SWCNTs) and were first reported in 1993 [4], while the ones with more than one well are multiwall carbon nanotubes (MWCNTs) and were first discovered in 1991 by Iijima [5] (Figure 1).

### Carbon nanotubes: structure and properties

Carbon can bond in different ways to construct structures with completely different properties. The sp^2^ hybridization of carbon builds a layered construction with weak out-of-plane bonding of the van der Waals form and strong in-plane bounds. A few to a few tens of concentric cylinders with the regular periodic interlayer spacing locate around ordinary central hollow and made MWCNTs. The real-space analysis of multiwall nanotube images has shown a range of interlayer spacing (0.34 to 0.39 nm) [9].

Depending on the number of layers, the inner diameter of MWCNTs diverges from 0.4 nm up to a few nanometers and outer diameter varies characteristically from 2 nm up to 20 to 30 nm. Both tips of MWCNT usually have closed and the ends are capped by dome-shaped half-fullerene molecules (pentagonal defects), and axial size differs from 1 μm up to a few centimeter. The role of the half-fullerene molecules (pentagonal ring defect) is to help in closing of the tube at the two ends.

On other hand, SWCNT diameters differ from 0.4 to 2 to 3 nm, and their length is typically of the micrometer range. SWCNTs usually can come together and form bundles (ropes). In a bundle structure, SWCNTs are hexagonally organized to form a crystal-like construction [3].

### MWCNT and SWCNT structure

**Figure 2:**
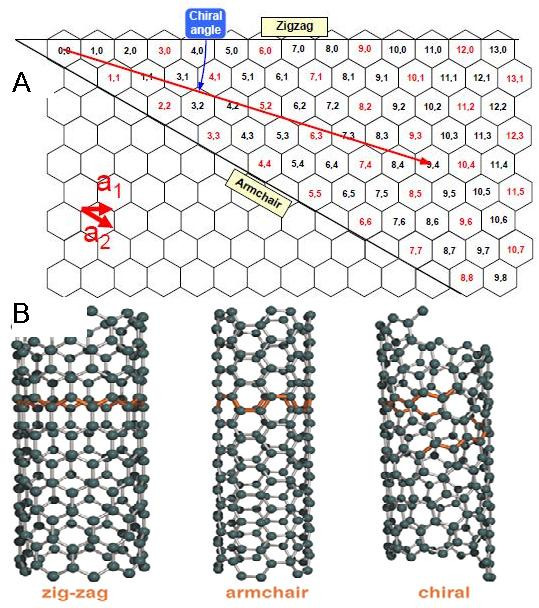
**Different forms of SWNTs. (A)** The chiral vector *C* also determines the tube diameter. **(B)** Models of three atomically perfect SWCNT structures [10].

Dependent on wrapping to a cylinder way, there are three different forms of SWCNTs such as armchair, chiral, and zigzag (Figure 2B). A SWCNT's structure is characterized by a pair of indices (*n*, *m*) that describe the chiral vector and directly have an effect on electrical properties of nanotubes. The number of unit vectors in the honeycomb crystal lattice of graphene along two directions is determined by the integers *n* and *m*. As a common opinion, when *m* = 0, the nanotubes are named zigzag nanotubes; when *n* = m, the nanotubes are named armchair nanotubes, and other state are called chiral.

The chiral vector *C* = *na*_1_ + *ma*_2_ (a_1_ and a_2_ are the base cell vectors of graphite) also determines the tube diameter *d*[4,5], and this vector finds out the direction of rolling a graphene sheet (Figure 2A). Therefore, the diameter of a carbon tube can be calculated by

$$d=\frac{a\sqrt{m^{2}+\mathrm{mn}+n^{2}}}{\pi }$$

where $a=1.42\times \sqrt{3}Å$ corresponds to the lattice constant in the graphite sheet.

When *n* − *m* is a multiple of 3, then the nanotube is described as ‘metallic’ or highly conducting nanotubes, and if not, then the nanotube is a semimetallic or semiconductor.

At all times, the armchair form is metallic, whereas other forms can make the nanotube a semiconductor.

Many parameters and vectors can have an effect on nanotube structures such as the following [6]:

(1) Translational vector = *T* = *t*1*a*1 + *t*2*a*2 » (*t*1, *t*2)

(2) Chiral vector = Ch = *na*1 + *na*2 » (*n*, *m*)

(3) Length of chiral vector = *L* = *a* √ (*n*^2^ + *m*^2^ + *n* * *m*), where *a* is the lattice constant

(4) Chiral angle = *cosθ* = (2*n* + *m*)/(2 * √ (*n*^2^ + *m*^2^ + *n* * *m*))

(5) Number of hexagons in the unit cell = *N* = (2 * (*n*^2^ + *m*^2^ + *n* * *m*)/*dR*)

(6) Diameter = *dt* = *L*/*π*

(7) Rotation angle of the symmetry vector = *ψ* = 2*π*/*N* (in radians)

(8) Symmetry vector = *R* = *pa*1 + *qa*2 » (*p*, *q*)

(9) Pitch of the symmetry vector = *τ* = ((*m* * *p*–*n* * *q*) * *T*)/*N*

Multiwalled carbon nanotubes can be formed in two structural models: Russian Doll model and Parchment model. When a carbon nanotube contains another nanotube inside it and the outer nanotube has a greater diameter than thinner nanotube, it is called the Russian Doll model. On other hand, when a single graphene sheet is wrapped around itself manifold times, the same as a rolled up scroll of paper, it is called the Parchment model. MWCNTs and SWCNTs have similar properties. Because of the multilayer nature of MWCNTs, the outer walls can not only shield the inner carbon nanotubes from chemical interactions with outside substances but also present high tensile strength properties, which do not exist in SWCNTs (or exist partially) [11] (Table 1).

**Table 1:** **Comparison between SWNT and MWNT**[4]

**SWNT**	**MWNT**
Single layer of graphene	Multiple layers of graphene
Catalyst is required for synthesis	Can be produced without catalyst
Bulk synthesis is difficult as it requires proper control over growth and atmospheric condition	Bulk synthesis is easy
Purity is poor	Purity is high
A chance of defect is more during functionalization	A chance of defect is less but once occurred it is difficult to improve
Less accumulation in the body	More accumulation in the body
Characterization and evaluation is easy	It has very complex structure
It can be easily twisted and is more pliable	It cannot be easily twisted

Since carbon nanotubes have the sp^2^ bonds between the individual carbon atoms, they have a higher tensile strength than steel and Kevlar. This bond is even stronger than the sp^3^ bond found in diamond. Theoretically, SWCNTs may really have a tensile strength hundreds of times stronger than steel.

Another amazing property of carbon nanotubes is also elasticity. Under high force and press sitting and when exposed to great axial compressive forces, it can bend, twist, kink, and finally buckle without damaging the nanotube, and the nanotube will return to its original structure, but an elasticity of nanotubes does have a limit, and under very physically powerful forces presses, it is possible to temporarily deform to shape of a nanotube. Some of the defects in the structure of the nanotube can weaken a nanotube's strength, for example, defects in atomic vacancies or a rearrangement of the carbon bonds.

Elasticity in both single and multiwalled nanotubes is determined by elastic modulus or modulus of elasticity [7]. The elasticity modulus of multiwall nanotubes (MWNTs) is analyzed with transmission electron microscopes (TEM). Scientists using the TEM measure and examine the thermal vibrations at both ends of the tubes. As a result of the strength of the atomic bonds in carbon nanotubes, they not only can withstand high temperatures but also have been shown to be very good thermal conductors. They can withstand up to 750°C at normal and 2,800°C in vacuum atmospheric pressures. The temperature of the tubes and the outside environment can affect the thermal conductivity of carbon nanotubes [8]. Some of the major physical properties of carbon nanotubes are summarized in Table 2.

**Table 2:** The physical properties of carbon nanotubes

**Physical properties**		**Values**
Equilibrium structure	Average diameter of SWNTs	1.2 to 1.4 nm
	Distance from opposite carbon atoms (line 1)	2.83 Å
	Analogous carbon atom separation (line 2)	2.456 Å
	Parallel carbon bond separation (line 3)	2.45 Å
	Carbon bond length (line 4)	1.42 Å
	C-C tight bonding overlap energy	Approximately 2.5 eV
	Group symmetry (10, 10)	C5V
	Lattice: bundles of ropes of nanotubes	Triangular lattice (2D)
Lattice constant		17 Å
Lattice parameter	(10, 10) Armchair	16.78 Å
	(17, 0) Zigzag	16.52 Å
	(12, 6) Chiral	16.52 Å
Density	(10, 10) Armchair	1.33 g/cm^3^
	(17, 0) Zigzag	1.34 g/cm^3^
	(12, 6) Chiral	1.40 g/cm^3^
Interlayer spacing:	(*n*, *n*) Armchair	3.38 Å
	(*n*, 0) Zigzag	3.41 Å
	(2*n*, *n*) Chiral	3.39 Å
Optical properties		
Fundamental gap	For (*n*, *m*); *n* − *m* is divisible by 3 [metallic]	0 eV
	For (*n*, *m*); *n* − *m* is not divisible by 3 [semiconducting]	Approximately 0.5 eV
Electrical transport		
	Conductance quantization	(12.9 k O )-1
	Resistivity	10-4 O -cm
	Maximum current density	1,013 A/m^2^
Thermal transport		
	Thermal conductivity	Approximately 2,000 W/m/K
	Phonon mean free path	Approximately 100 nm
	Relaxation time	Approximately 10 to 11 s
Elastic behavior		
	Young's modulus (SWNT)	Approximately 1 TPa
	Young's modulus (MWNT)	1.28 TPa
	Maximum tensile strength	Approximately 100 GPa

### Synthesis

There are several techniques that have been developed for fabricating CNT structures which mainly involve gas phase processes. Commonly, three procedures are being used for producing CNTs: (1) the chemical vapor deposition (CVD) technique [12,13], (2) the laser-ablation technique [3,9], and (3) the carbon arc-discharge technique [14-16] (Table 3). High temperature preparation techniques for example laser ablation or arc discharge were first used to synthesize CNTs, but currently, these techniques have been substituted by low temperature chemical vapor deposition (CVD) methods (<800°C), since the nanotube length, diameter, alignment, purity, density, and orientation of CNTs can be accurately controlled in the low temperature chemical vapor deposition (CVD) methods [17].

**Table 3:** Summary and comparison of three most common CNT synthesis methods

**Method**	**Arc discharge**	**Laser ablation**	**CVD**
Yield rate	>75%	>75%	>75%
SWNT or MWNT	Both	Both	Both
Advantage	Simple, inexpensive, high-quality nanotubes	Relatively high purity, room-temperature synthesis	Simple, low temperature, high purity, large-scale production, aligned growth possible
Disadvantage	High temperature, purification required, tangled nanotubes	Method limited to the labscale, crude product purification required	Synthesized CNTs are usually MWNTs, defects

#### Electric arc discharge

Arc-discharge technique uses higher temperatures (above 1,700°C) for CNT synthesis which typically causes the expansion of CNTs with fewer structural defects in comparison with other methods. The most utilized methods use arc discharge between high-purity graphite (6 to 10-mm optical density (OD)) electrodes usually water-cooled electrodes with diameters between 6 and 12 mm and separated by 1 to 2 mm in a chamber filled with helium (500 torr) at subatmospheric pressure (helium can be replaced by hydrogen or methane atmosphere) [10]. The chamber contains a graphite cathode and anode as well as evaporated carbon molecules and some amount of metal catalyst particles (such as cobalt, nickel, and/or iron). Direct current is passed through the camber (arcing process), and the chamber is pressurized and heated to approximately 4,000 K. In the course of this procedure and arcing, about half of the evaporated carbon solidifies on the cathode (negative electrode) tip, and a deposit forms at a rate of 1 mm/min which is called ‘cylindrical hard deposit or cigar-like structure’, whereas the anode (positive electrode) is consumed. The remaining carbon (a hard gray shell) deposited on the periphery and condenses into ‘chamber soot’ nearby the walls of the chamber and ‘cathode soot’ on the cathode. The inner core, cathode soot and chamber soot, which are dark and soft, yield either single-walled or multiwalled carbon nanotubes and nested polyhedral graphene particles. By using scanning electron microscopy (SEM), two different textures and morphologies can be observed in studying of the cathode deposit; the dark and soft inner core deposits consist of bundle-like structures, which contain randomly arranged nanotubes and the gray outer shell, which is composed of curved and solid grapheme layers.

In the arc discharge deposition and synthesis of CNTs, there are two main different ways: synthesis with use of different catalyst precursors and without use of catalyst precursors. Generally, synthesis of MWNTs could be done without use of catalyst precursors but synthesis of single-wall nanotubes (SWNTs) utilizes different catalyst precursors and, for expansion in arc discharge, utilizes a complex anode, which is made as a composition of graphite and a metal, for example, Gd [11], Co, Ni, Fe, Ag, Pt, Pd, etc., or mixtures of Co, Ni, and Fe with other elements like Co-Pt, Co-Ru [18], Ni-Y, Fe-Ni, Co-Ni, Co-Cu, Ni-Cu, Fe-No, Ni-Ti, Ni-Y, etc. Studies have shown Ni-Y-graphite mixtures can produce high yields (<90%) of SWNTs (average diameter of 1.4 nm) [19], and nowadays, this mixture is used worldwide for creation of SWNTs in high yield. The main advantage of arc-discharge technique is ability and potential for production of a large quantity of nanotubes. On the other hand, the main disadvantage of this method is relatively little control over the alignment (i.e., chirality) of the created nanotubes, which is important for their characterization and role. Additionally, because of the metallic catalyst needed for the reaction, purification of the obtained products is essential.

#### Laser ablation method

By using of high-power laser vaporization (YAG type), a quartz tube containing a block of pure graphite is heated inside a furnace at 1,200 ± C, in an Ar atmosphere [12]. The aim of using laser is vaporizing the graphite within the quartz. As described about the synthesis of SWNT by using arc-discharge method, for generating of SWNTs, using the laser technique adding of metal particles as catalysts to the graphite targets is necessary. Studies have shown the diameter of the nanotubes depends upon the laser power. When the laser pulse power is increased, the diameter of the tubes became thinner [13]. Other studies have indicated ultrafast (subpicosecond) laser pulses are potential and able to create large amounts of SWNTs [14]. The authors revealed that it is now promising to create up to 1.5 g/h of nanotube material using the laser technique.

Many parameters can affect the properties of CNTs synthesized by the laser ablation method such as the structural and chemical composition of the target material, the laser properties (peak power, cw versus pulse, energy fluence, oscillation wavelength, and repetition rate), flow and pressure of the buffer gas, the chamber pressure and the chemical composition, the distance between the target and the substrates, and ambient temperature. This method has a potential for production of SWNTs with high purity and high quality. The principles and mechanisms of laser ablation method are similar to the arc-discharge technique, but in this method, the needed energy is provided by a laser which hit a pure graphite pellet holding catalyst materials (frequently cobalt or nickel).

The main advantages of this technique consist of a relatively high yield and relatively low metallic impurities, since the metallic atoms involved have a tendency to evaporate from the end of the tube once it is closed. On other hand, the main disadvantage is that the obtained nanotubes from this technique are not necessarily uniformly straight but instead do contain some branching.

Unfortunately, the laser ablation method is not economically advantageous because the procedure encompasses high-purity graphite rods, the laser powers required are great (in some cases two laser beams are required), and the quantity of nanotubes that can be synthesized per day is not as high as arc-discharge technique.

#### Chemical vapor deposition

One of standard methods for production of carbon nanotubes is chemical vapor deposition or CVD. There are many different types of CVD such as catalytic chemical vapor deposition (CCVD)—either thermal [20] or plasma enhanced (PE) oxygen assisted CVD [5], water assisted CVD [21-23], microwave plasma (MPECVD) [24], radiofrequency CVD (RF-CVD) [25], or hot-filament (HFCVD) [26,27]. But catalytic chemical vapor deposition (CCVD) is currently the standard technique for the synthesis of carbon nanotubes.

This technique allows CNTs to expand on different of materials and involves the chemical breakdown of a hydrocarbon on a substrate. The main process of growing carbon nanotubes in this method as same as arc-discharge method also is exciting carbon atoms that are in contact with metallic catalyst particles.

For all intents and purposes, tubes are drilled into silicon and also implanted with iron nanoparticles at the bottom. After that, a hydrocarbon such as acetylene is heated and decomposed onto the substrate. Since the carbon is able to make contact with the metal particles implanted in the holes, it initiates to create nanotubes which are a ‘template’ from the shape of the tunnel. With using of these properties, the carbon nanotubes can grow very well aligned and very long, in the angle of the tunnel. In CVD processing, a layer of metal catalyst particles prepare and process a substrate at approximately 700°C. Most commonly, metal catalyst particles are nickel, cobalt [28], iron, or a combination [29]. The aim of using the metal nanoparticles in combination with a catalyst support such as MgO or Al2O3 is to develop the surface area for higher by-product of the catalytic reaction of the pure carbon with the metal particles. In the first step of nanotube expansion, two types of gases fueled the reactor (the most widely used reactor is fluidized bed reactor [30,31]): a carbon-containing gas (such as ethylene, acetylene, methane, or ethanol) and a process gas (such as nitrogen, hydrogen, or ammonia). At the surface of the catalyst particle, the carbon-containing gas is broken apart and so the carbon became visible at the edges of the nanoparticle where the nanotubes can produce. This mechanism is still under discussion [32]. Studies have shown the conventionally accepted models are base growth and tip growth [33]. Depending on the adhesion and attachment between the substrate and the catalyst particle, the catalyst particles can remain at the nanotube base or nanotube during growth and expansion [34].

As compared with laser ablation, CCVD is an economically practical method for large-scale and quite pure CNT production and so the important advantage of CVD are high purity obtained material and easy control of the reaction course [35].

### Nanotube purification

Depending on technique of carbon nanotube synthesis, there are many different methods and procedure for purification. All purification procedures have the following main steps: deletion of large graphite particles and aggregations with filtration, dissolution in appropriate solvents to eliminate catalyst particles (concentrated acids as solvent) and fullerenes (use of organic solvents), and microfiltrations and chromatography to size separation and remove the amorphous carbon clusters [35]. Purification of MWNTs produced by arc-discharge techniques can be done by using oxidation techniques which can take apart MWNTs from polyhedral graphite-like particles [10].

The main disadvantages of this method are low purity, high destroying rate of starting materials (95%), as well as high reactivity of the remaining nanotubes at end of process due to existence of dangling bonds (an unsatisfied valence) [36] and for elimination of such dangling bonds is necessary to use high-temperature annealing (2,800 ± C).

The nondestructive methods for separating CNTs couple well-dispersed colloidal suspensions of tubes/particles with materials which prevent aggregation such as surfactants, polymers, or other colloidal particles [37]. The other method as aim of size exclusion nanotubes uses size exclusion chromatography and porous filters [37] as well as ultrasonically assisted microfiltration which purifies SWNTs from amorphous carbon and catalytic particles [38].

Studies have shown the boiling of SWNTs in nitric acid [39] or hydrofluoric acid [40] aqueous solutions for purification of SWNTs and removing amorphous carbon and metal particles as an efficient and simple technique.

For the purification of carbon tubules, scientist prefers to use sonication of nanotube in different media and afterward thermal oxidation of SWNT material (at 470°C) as well as hydrochloric acid treatments [41]. Another way for oxidizing unsatisfied carbonaceous particles is use of gold clusters (OD 20 nm) together with the thermal oxidation of SWNTs at 350°C [42].

Huang et al. introduce a new way for separation of semiconducting and metallic SWNTs by using of size exclusion chromatography (SEC) of DNA-dispersed carbon nanotubes (DNA-SWNT), which have the highest resolution length sorting [43]. The density-gradient ultracentrifugation has been used for separation of SWNT based on diameter [44]. Combination of ion-exchange chromatography (IEC) and DNA-SWNT (IEC-DNA-SWNT) has also been used for purification of individual chiralities. In this process, specific short DNA oligomers can be used to separate individual SWNT chiralities. Scientists have used fluorination and bromination processes as well as acid treatments of MWNT and SWNT material with the aims of purifying, cutting, and suspending the materials uniformly in certain organic solvents [45,46].

As discussed above, depending on nanotube synthesis way, there are many different methods for purification of carbon nanotubes, and therefore, existence of methods which are single-step processes and unaffected on properties of carbon nanotube products is essential for producing clean nanotubes and should be targeted in the future.

### Biomedical applications

The properties of nanotubes are certainly amazing; in the last few years, many studies have suggested potential applications of CNTs and have shown innumerable applications that could be promising when these newly determined materials are combined with typical products [36,47-51]. Production of nanorods using CNTs as reacting templates [51-55].

Applications for nanotubes encompass many fields and disciplines such as medicine, nanotechnology, manufacturing, construction, electronics, and so on. The following application can be noted: high-strength composites [54,56-61], actuators [62], energy storage and energy conversion devices [63], nanoprobes and sensors [61], hydrogen storage media [64], electronic devices [65], and catalysis [66]. However, the following sections detail existing applications of CNTs in the biomedical industry exclusively. Before use of carbon nanotube in biological and biomedical environments, there are three barriers which must be overcome: functionalization, pharmacology, and toxicity of CNTs. One of the main disadvantages of carbon nanotubes is the lack of solubility in aqueous media, and to overcome this problem, scientists have been modifying the surface of CNTs, i.e., fictionalization with different hydrophilic molecules and chemistries that improve the water solubility and biocompatibility of CNT [67].

Another barrier with carbon nanotube is the biodistribution and pharmacokinetics of nanoparticles which are affected by many physicochemical characteristics such as shape, size, chemical composition, aggregation, solubility surface, and fictionalization. Studies have shown that water-soluble CNTs are biocompatible with the body fluids and do not any toxic side effects or mortality.

Another important barrier is toxicity of CNTs. Generally, the combination of the high surface area and the intrinsic toxicity of the surface can be responsible for the harmful effects of nanoparticles.

The toxicity of CNTs can be affected by the size of nanotubes. The particles under 100 nm have potential harmful properties such as more potential toxicity to the lung, escape from the normal phagocytic defenses, modification of protein structure, activation of inflammatory and immunological responses, and potential redistribution from their site of deposition.

### Artificial implants

Nanomaterials show probability and promise in regenerative medicine because of their attractive chemical and physical properties [68]. Generally, reject implants with the postadministration pain, and to avoid this rejection, attachment of nanotubes with proteins and amino acids has been promising. Carbon nanotube, both single and multi-WNT, can be employed as implants in the form of artificial joints and other implants without host rejection response. Moreover, because of unique properties such as high tensile strength, CNTs can act as bone substitutes and implants if filled with calcium and shaped/arranged in the bone structure [69,70].

It has been investigated the cellular adhesion and proliferation can enhance with SWCNT and MWCNT composites, and therefore, these nanotubes have been integrated into natural and synthetic materials to generate nanocomposites. Some nanotube applications as artificial implants are summarized in Table 4.

**Table 4:** Application of nanotube as artificial implants

**CNT type**	**Natural or synthetic materials type**	**Cell or tissue type**	**Properties**	**Reference(s)**
Porous SWCNT	Polycarbonate membrane	Osteoblast-like cells	Increase lamellipodia (cytoskeletal) extensions, and lamellipodia extensions	[71]
SWCNT-incorporated	Chitosan scaffolds	C2Cl2 cells /C2 myogenic cell line	Cell growth improvement	[72]
MWCNT	Collagen sponge honeycomb scaffold	MC3T3-E1 cells, a mouse osteoblast-like cell line	Increase cellular adhesion and proliferation	[73]
MWCNT	Polyurethane	Fibroblast cells	Enhance interactions between the cells and the polyurethane surface	[74]
SWCNT	Alginate	Rat heart endothelial cell	Enhance cellular adhesion and proliferation	[75]
MWCNT	Poly(acrylic acid)	Human embryonic stem cells	Increase cellular differentiation toward neurons	[76]
SWCNT	Propylene fumarate	Rabbit tibia	Support cell attachment and proliferation	[77]

#### Tissue engineering

The aim of tissue engineering is to substitute damaged or diseased tissue with biologic alternates that can repair and preserve normal and original function. Major advances in the areas of material science and engineering have supported in the promising progress of tissue regenerative medicine and engineering. Carbon nanotubes can be used for tissue engineering in four areas: sensing cellular behavior, cell tracking and labeling, enhancing tissue matrices, and augmenting cellular behavior [78]. Cell tracking and labeling is the ability to track implanted cells and to observe the improvement of tissue formation in vivo and noninvasively. Labeling of implanted cells not only facilitates evaluating of the viability of the engineered tissue but also assists and facilitates understanding of the biodistribution, migration, relocation, and movement pathways of transplanted cells. Because of time consuming and challenge of handling in using of traditional methods such as flow cytometry, noninvasive methods are incoming popular methods. It is shown carbon nanotubes can be feasible as imaging contrast agents for magnetic resonance, optical, and radiotracer modalities.

Another important application of carbon nanotubes in tissue engineering is its potential for measure of biodistribution and can also be modified with radiotracers for gamma scintigraphy. Singh et al. bound SWNTs with [79]. In and administered to BALB/c mice to evaluate the biodistribution of nanotubes [80]. The design of better engineered tissues enhances and facilitates with the better monitor of cellular physiology such as enzyme/cofactor interactions, protein and metabolite secretion, cellular behavior, and ion transport. Nanosensors possibly will be utilized to make available constant monitoring of the performance of the engineered tissues. Carbon nanotubes present numerous popular features that make them ideal elements for nanosensors including their large surface area and capacity to immobilize DNA or other proteins, and electrical properties. The carbon nanotube has unique electronic structures which as carbon nanotube electrochemical sensor probability makes simpler the investigation of redox-active proteins and amino acids allowing cell monitoring in engineered tissues. In one study, MWNTs were conjugated with platinum microparticles and were able to sense thiols including amino acids such as glutathione and L-cysteine in rat [81].

The matrix of cells plays an important role in tissue engineering. While accepted synthetic polymers, for example, PLGA and PLA have been employed for tissue engineering, they lack the required mechanical strength and cannot simply be functionalized in contradiction of carbon nanotubes which can be voluntarily functionalized. Thus, carbon nanotubes have potential for use as tissue scaffolds and can provide the required structural reinforcement, but the main disadvantage of carbon nanotubes is that they are not biodegradable. Combination of polymer by dissolving a desired portion of carbon nanotubes into a polymer, significant enhancements in the mechanical strength of the composite has been detected. MWNTs combined with chitosan illustrated significant advancement in mechanical properties compared with only chitosan [82]. The SWNT blended collagen improves smooth muscle cell growth [83-89].

### Cancer cell identification

Nanodevices are being created that have a potential to develop cancer treatment, detection, and diagnosis. Nanostructures can be so small (less than 100 nm) that the body possibly will clear them too quickly for them to be efficient in imaging or detection and so can enter cells and the organelles inside them to interact with DNA and proteins. Castillo et al., by using a peptide nanotube-folic acid modified graphene electrode, improve detection of human cervical cancer cells overexpressing folate receptors [90-96].

Since a large amount of cancers are asymptomatic throughout their early stage and distinct morphologic modifications are absent in the majority of neoplastic disorders in early stage, consequently traditional clinical cancer imaging methods, for example, X-ray, CT, and MRI, do not acquire adequate spatial resolution for detection of the disease in early stage. The imaging studies with SWCNTs have thrived over the past few years. Hong et al. [97] evaluated the molecular imaging with SWNTs and evaluated the combined Gd3 + -functionalized SWCNTs when applied to MRI, and high resolution and good tissue penetration were achieved.

Combination of radioisotopes labeled SWCNTs with radionuclide based imaging techniques (PET and SPECT) can improve the tissue penetration, sensitivity, and medium resolution.

There are many characteristic protein biomarkers which often are overexpressed in cancer cells, and they provide an opening gate for early diagnosis, prognosis, maintaining surveillance following curative surgery, monitoring therapy in advanced disease, and predicting therapeutic response. Many important tumor markers have been extensively applied and used in the diagnosis of hepatocellular carcinoma, colorectal cancer, pancreatic cancer, prostate cancers, epithelial ovarian tumor such as carbohydrate antigen 19-9 (CA19-9), alpha-fetoprotein (AFP), carcinoembryonic antigen (CEA), carcinoma antigen 125 (CA125), human chorionic gonadotropin (hCG), and prostate-specific antigen (PSA). Some of the cancer biomarkers which are detected by CNT-based detection systems are summarized in Table 5.

**Table 5:** Example of detection of cancer biomarker by carbon nanotubes

**Carbon nanotube**	**Biomarker**	**Form of cancer**	**Reference**
P-type carbon nanotubes	Prostate-specific antigen (PSA)	Prostate cancer	[98]
Multilabel secondary antibody-nanotube bioconjugates	Prostate-specific antigen (PSA)	Prostate cancer	[99]
Microelectrode arrays modified with single-walled carbon nanotubes (SWNTs)	Total prostate-specific antigen (T-PSA)	Prostate cancer	[99]
Multiwalled carbon nanotubes-thionine-chitosan (MWCNTs-THI-CHIT) nanocomposite film	Chlorpyrifos residues	Many forms	[100]
Carbon nanomaterial	Carcinoma antigen-125 (CA125)	Carcinoma	[101]
MWCNT-platinum nanoparticle-doped chitosan (CHIT)	AFP	Many forms	[102]
Poly-l-lysine/hydroxyapatite/carbon nanotube (PLL/HA/CNT) hybrid nanoparticles	Carbohydrate antigen 19–9 (CA19-9)	Many forms	[103]
MWCN-polysulfone (PSf) polymer	Human chorionic gonadotropin (hCG)	Many forms	[104]
Multiwalled carbon nanotube-chitosan matrix	Human chorionic gonadotropin (hCG)	Many forms	[105]
MWCNT-glassy carbon electrode (GCE)	Prostate-specific antigen (PSA)	Prostate cancer	[106]
Nanoparticle (NP) label/immunochromatographic electrochemical biosensor	Prostate-specific antigen (PSA)	Prostate cancer	[107]
SWNT-horseradish peroxidase (HRP)	Prostate-specific antigen (PSA)	Prostate cancer	[107]
Carbon nanotube field effect transistor (CNT-FET)	Prostate-specific antigen (PSA)	Prostate cancer	[108]
Carbon nanoparticle (CNP)/poly(ethylene imine) (PEI)-modified screen-printed graphite electrode (CNP-PEI/SPGE)	Carcinoembryonic antigen (CEA),	Urothelial carcinoma	[109]
Tris(2,2′-bipyridyl)cobalt(III) (Co(bpy)33+)- MWNTs-Nafion composite film	Carcinoma antigen-125 (CA125)	Carcinoma	[79]
Gold nanoparticles and carbon nanotubes doped chitosan (GNP/CNT/Ch) film	Alpha-fetoprotein (AFP)	Many forms	[110]
Multiple enzyme layers assembled multiwall carbon nanotubes (MWCNTs)	Alpha-fetoprotein (AFP)	Many forms	[111]

### Drug and gene delivery by CNTs

There are many barriers with conventional administration of chemotherapeutic agents such as lack of selectivity, systemic toxicity, poor distribution among cells, limited solubility, inability of drugs to cross cellular barriers, and lack of clinical procedures for overcoming multidrug resistant (MDR) cancer [112,113]. Researchers have introduced a wide range of different types of drug delivery systems to overcome these problems such as polymers, silica nanoparticles, quantum dots, emulsions, dendrimers, liposomes, molecular conjugates, and micelles [114]. As mentioned above, CNTs have the unique properties such as ultrahigh surface area which make them as promising potential for delivery of drugs, peptides, and nucleic acids (Table 6). The specific drug or gene can be integrated to walls and tips of CNTs and recognize cancer-specific receptors on the cell surface, by these means CNTs can cross the mammalian cell membrane by endocytosis or other mechanisms [115] and carry therapeutic drugs or genes more safely and efficiently in the cells that are previously inaccessible [116]. More recently, researchers have developed a novel and more efficient SWNT-based tumor-targeted drug delivery system (DDS) which consists of tumor-targeting ligands, anticancer drugs, and functionalized SWNTs. If this system interacts with cancer cells, then it can induce receptor-mediated endocytosis by recognizing cancer-specific receptors on the surface of cancer cells and so efficiently and specifically release chemotherapeutic agents.

**Table 6:** Example of drugs and nucleic acids which were delivered by carbon nanotubes

**Drug/nucleic acid**	**CNT type**	**Cell or tissue**	**Properties**	**Reference**
Taxoid	SWNTs	Leukemia	High potency toward specific cancer cell lines	[116]
Doxorubicin	SWNTs	Colon cancer	Efficiently taken up by cancer cells, then translocates to the nucleus while the nanotubes remain in the cytoplasm	[113,114]
Cisplatin	SWNTs	Squamous carcinoma	Rapid regression of tumor growth	[117]
Cisplatin	SWNTs	Nasopharyngeal epidermoid carcinoma, etc.	High and specific binding to the folate receptor (FR) for the SWNT-1 conjugate	[118]
Doxorubicin	SWNTs	Breast cancer Glioblastoma	Show that large surface areas on single-walled carbon nanotubes (SWNTs)	[119]
Doxorubicin	SWNTs	Cervical carcinoma	Increase nuclear DNA damage and inhibit the cell proliferation	[115]
Radionuclide	SWNTs	Burkitt lymphoma	The selective targeting of tumor in vitro and in vivo	[120]
Paclitaxel	SWNTs	Breast cancer	High treatment efficacy, minimum side effects	[121]
siRNA	SWNTs	Tumor cells both in vitro and in vivo mouse models	Increase suppression of tumor growth	[122]
Toxic siRNA sequence (siTOX)	Functionalized MWNTs	Human lung xenograft model	Significant tumor growth inhibition	[123]
siRNA	SWNT	Human neuroblastoma	Enhance the efficiency of siRNA-mediated gastrin-releasing peptide receptor (GRP-R) gene silencing	[124]
*SOCS1*siRNA	sWNT	Dendritic cells (DCs)	Reduced *SOCS1* expression and retarded the growth of established B16 tumor in mice	[125]

## Conclusions

Nanomaterials explain probability and promise in regenerative medicine for the reason that of their attractive chemical and physical properties.

Carbon nanotubes (purified/modified) have a high potential of finding unique applications in wide areas of medicine. Moreover, the encapsulation of other materials in the carbon nanotubes would open up a prospect for their bioapplications in medicine.

There remains amount of essential issues that require to be resolved, on the other hand, such as homogeneity of the material that contains wide distribution of the nanotube's diameters, unlike nanostructures, presence of residual metals; division of the individual nanotubes; and a sensitivity to the different gases and species [126-139].

## Competing interests

The authors declare that they have no competing interests.

## Authors’ contributions

AE, HK, and NZ conceived of the study and participated in its design and coordination. AA, MK, and SWJ assisted in the numerical calculations. HD, MA, and YH participated in the sequence alignment and drafted the manuscript. SWJ supervised the whole study. All authors read and approved the final manuscript.
